# Preparing for online interviews during Covid-19: the intricacies of technology and online human interaction

**DOI:** 10.1007/s43545-022-00498-2

**Published:** 2022-09-26

**Authors:** Şahizer Samuk Carignani, Sandra Burchi

**Affiliations:** 1 LYNX Research Center, IMT School for Advanced Studies, Lucca, Italy; 2 UBIQUAL, Department of Political Science, University of Pisa, Via Filippo Serafini, 3, 56126 Pisa, PI Italy

**Keywords:** Online interviews, Qualitative research, Virtual fieldwork, Highly skilled, COVID-19

## Abstract

How can we guarantee that “extracting data” is realised most respectfully and reciprocally online? How can we receive the most relevant responses from the interviewee in online interviews? These questions have been even more pertinent during the Covid-19 pandemic. In this paper, we aim to demonstrate how the preparation of the research process that involves online interviews with highly skilled Italians abroad, functions when a group of social scientists come together, and take decisions on criteria and modality of virtual fieldwork. The intricacies of the online interviews are numerous. Yet, there is a research gap regarding the details of the process of conducting them. We find that the periods before, during and after online interviews indicate a whole learning process, which is neglected in the current literature. Hence, we argue that organisation, use of time, density of the themes, mindfulness, synchronisation and handling of sensitive issues are the main tenets of the art of doing online interviews. In this paper, we explore and explain each aspect, also in a chronological manner, benefiting from the previous literature and contributing to research with our anthropological and sociological insights about using technology whilst conducting online interviews with highly skilled Italians abroad.

## Introduction

During the first and second phase of Covid-19, we discussed as the research team on how to conduct online interviews with Italian emigrants in the most efficient way. However, as we have deliberated, many complications might arise from online interviews. For this reason, we conducted a pilot interview as the authors of this paper. In this paper, we also included all the planning and discussion process of our preparation process. During the preparation process for our pilot interview, my colleague and I started the online interview using the software of Microsoft teams, and the interview lasted almost 2 h.^1^ The pilot interview we realised online (as researchers and experts on mobility and migration) is not the central aim of writing this paper. However, it has provided us with the motivation to elaborate on the pros, cons and complications of online interviews emanating from this micro experience. Whilst we describe the pilot interview, we also focus on the set of interviews that we conducted online in order to explore the way in which online interviews with the highly skilled and spatially mobile people function and which are the elements that the researchers should consider before starting the virtual fieldwork.

The reason that this research paper is acute and timely is that the Covid-19 has affected our lives intensely during the year 2020 (even into 2021) and most of the plans to do fieldwork in a pre-planned place (town, city, country) were postponed. Some of the researchers had to do online interviews, although that was not the initial plan. Still, interviews are indispensable to the qualitatively minded researchers. The fact that we cannot do face-to-face interviews does not mean that we will discard the interviews totally and rely only on secondary resources online. Hence, with this paper, we hope to provide a contribution to the literature with our suggestions to improve the online interview results.

It is observed that most of the time, the researchers are aware of their time limitations, and they might want to do a few interviews leaning on the biographical elements. On the contrary, they might also decide to conduct numerous interviews to make further generalisations and understand multiple sociological dimensions (such as age, gender, work choices, educational background of the family, past spatial mobility experiences, international programmes that support spatial mobility, links between spatial mobility and migration), despite feeling the pressure of the lengthy times that transcription and analysis require. In the former case, the researchers will be criticised for paying too much attention to the biographies of the interviewees and not reaching a generalisable and saturated number. In the latter case, it might seem that the quantification makes end-result seem to be more important than the process of conducting in-depth interviews that allow the researcher to capture the biographical elements. The discussions of the researchers regarding how the semi-structured online interview questions shall be prepared have given us ample space to think and introduce the idea to write about diverse approaches to semi-structured and unstructured interviews online.

Synchronous interviews (online) can bring “opportunities for real-time responses from participants as well as a high level of participant involvement” (James and Busher 2012, p. 179). The same is true for face-to-face interviews. However, when it comes to geographically dispersed populations the face-to-face interviews are impossible to conduct (Cater 2011). In our case, the interviews had to be online due to two reasons: geographically dispersed skilled Italians and Covid-19 restrictions. However, it can be noted that the online interviews have different shortcomings. For instance, the possibility of deep listening is much harder compared to in-person interviews (‘t Hart 2021). Further, the uncertainty remains as the screen remains a barrier (Hine 2015) to understand the body language to a great extent. Additionally, the screen can turn the subjects into being “flat” rather than revealing all their specific characters (Adams-Hutcheson and Longhurst 2017). In line with these remarks, James and Busher (2016, pp. 8–9) categorise the advantages and disadvantages of online interviews as seen below:

**Table 1:** Advantages and disadvantages of online interviews explained by James and Busher (2016)

Advantages	Disadvantages
Savings of costs Location, geography and travel Flexibility Venue Engagement in the online interview Speed	Time lags in the online conversation Distracted participants Participants’ interest and motivations Language use Technical competence and failing technology Access Identity verification Absence of verbal cues

*Source* James and Busher (2016, pp. 8–9)

With the interviews in general the researcher finds him or herself at the centre of a very sensitive and stratified context, which generates rich outcomes in terms of social research (Cardano 2018). Despite the fact that the online interviews are said to be “alienating”, to use internet as an everyday experience and connect with people online is a practice intertwined with everyday life and social activities (Woolgar 2002).

In this paper, first, we have a background overview of our thought process and a thorough literature review that focuses on the online (internet-based or computer-mediated) interviews with advantages, disadvantages and complications that can be fruitful or counter-productive; second, we have three main sections: before the interview (preparing the questions, choosing the tools and sampling), during the interview (unstructured interview online, voice and appearance, perception of time) and after the interview (observation of the interview with some suggestions). Third, we explain how we conducted 51 interviews (with highly skilled Italians abroad) online and what kind of novel experience and scientific information regarding methodologies can be derived from this experience. Finally, we have concluding thoughts based on our experience linking our research with the previous literature review whilst suggesting alternative research agendas for future research on interviews in general and online interviews in particular.

## Background of the research context

The preparation process for the online interviews with the highly skilled migrants abroad require diligence to the extent that the same rigour is expected from face-to-face interviews. In addition to the preparations in face-to-face interviews, one has to choose the software to conduct the interviews. This decision is not immediate as with Covid-19 the number of software we have been using have augmented (Microsoft Teams, Zoom, Skype, Google meets, etc.). Nevertheless, the sociological dimensions of this research reveal that the online interviews with highly skilled migrants have its advantages. As indicated in the introduction section, there are limitations to the online interviews: in order to conduct successful online interviews, “high speed internet access and computer literacy of all parties” (Janghorban et al. 2014) are essential. Since our interviewees are skilled ones, who use software to communicate with their colleagues and also their families in their daily lives, our interviewees were not affected by these limitations. Moreover, the interviewees have had previous experience abroad and have led transnational lives (Tables 1, 2, 3).

**Table 2:** Online interviews in comparison with the face-to-face interviews

Themes	Online interviews	There is not much difference between these two interviewing methods
Connection	Connection might be a problem and the video might freeze	Answers obtained via online interviews are quite the same as the face-to-face interviews (Denscombe 2003) Pauses, repetitions and recasts under conditions of face to face and online interviews do not differ significantly (Cabaroglu et al. 2010) The online interviews should not be considered as an easy option (James and Busher 2009, p. 9) Online interviews are not as simple as “point and click” (Cooper 2009) Online exchanges can be both liberating and limiting (Walther 1996)
Security and privacy	Identity verification can be an issue (Chen and Hinton 1999)	
Vulnerability of certain groups	Certain groups might be hard to reach (O’Connor et al. 2008)	
Costs	Internationalised research without the travel costs (O’Connor et al. 2008). Financial costs minimised via skype (Deakin and Wakefield 2014, p. 608)	
Nonverbal and verbal clues	Lack of nonverbal cues in online interviews (Hay-Gibson 2009)	
Ethical aspects	The ethical aspect of online interviews is “work in progress” (Madge 2010)	
Extraordinary circumstances such as pandemic times	During the Covid-19 online interviews has provided great opportunities	
Geographical aspect	When the research population is geographically dispersed (Sedgwick and Spiers 2009) it is easier	
Time wise	For those who work, to do online interviews in the evening is very convenient (Deakin and Wakefield 2014, p. 609)	
Listening is difficult due to various reasons	Listening is difficult with online interviews (‘t Hart 2021) Despite synchronous co presence it is observed, a level of uncertainty remains and the screen is a barrier to accurate interpretations of emotions and body language (Hine 2015). The same issue is defined as uncertainty by ‘t Hart (2021). ‘t Hart (2021) also it means that there is co presence but physical separation	
Absentees	Absentees (Mann and Stewart 2003). No shows can occur (Daekin and Wakefield 2014)	
Power imbalance is less	Destabilise power imbalance between the interviewee and the interviewer (Hanna 2012) Greater flexibility in organising a suitable time (Sturges and Hanrahan 2004)	
Flatness and dimensions	Turns people into flat characters (Adams‐Hutcheson and Longhurst 2017)	
To escape is easy for the research participant	Withdrawal is very easy with one click (Janghorban et al. 2014)	
Possibility of lost data	Video is slower than real time and potentially lost data as a result of technological failure (Sullivan 2012)	

*Source* Own elaboration

**Table 3:** Before, during and after the interviews: procedures and processes

Before	During	After	Learning
Ethical Board meeting and preparation of the documents to have the permit to do interviews online Preparing the consent forms for the interviewees Choosing the sample Deciding on how to reach the sample Deciding on the number of the interviews (open-ended discussion) Deciding on the type of interview: structured, non-structured or semi-structured Choosing the software Prepare the google forms to reach the sample Preparing the interview questions Preparing the matrix of codes, themes central to the main research question Do a pilot interview (or a couple of interviews) to try the questions and gain innovative insights Doublecheck the interview questions Plan B: Change the numbers of the interviewees; change the strategy if the google forms do not generate the representative sample amongst those who answer Being an insider and outsider to the research: thinking about the philosophical and sociological dimensions of being “highly skilled” emigrant or spatially mobile person Find a distraction-free place	Both in pilot and real interviews it is very central to introduce the researchers, the theme of the research, why the research is being done, who guides the research process and which institutions support the research A good introduction or a small talk together with the introduction can melt the ice Pilot interview (unstructured): Spontaneous speaking, silences and the effort to fill the silences Project interviews (semi-structured): One researcher took notes, the other one listened and took notes from time to time. One researcher asked the questions whilst the other interrupted if there was a theme of interest Voice can be lost from time to time as there are time shifts in the online interviews The appearance is just a squared version of the real person, so some nonverbal clues are lost Awareness of time is important (time flies the same way it does with the online interviews) Loss of synchronisation despite the good quality of the internet connection It is possible to observe partially the feelings of the interviewees; during the pilot interview, due to professionalism it was hard to notice the feelings of the other person	Organising the notes Discussing the interview results: what is new and what is interesting? The themes come to the fore with each interview added Memo writing (writing together or alone, and then comparing the notes) Discussion: What can be done better? Sending a “thank you” message to the interviewee for their time and attention Transcriptions and preliminary analysis of the main themes that are predominant in each interview; novel themes that have not been underlined in the literature before Coding process in Atlas.ti Writing papers	Observation of the pilot interview(s) Organisation Time awareness Density of themes Building rapport Mindfulness Synchronisation Sensitive issues

*Source* Own elaboration

From a sociological and cultural point of view, they already have a higher educational attainment including the social and cultural capital (Bourdieu 1986) that allow them to communicate online for work and private purposes. Therefore, factors such as unfamiliarity with online communication tools that could have affected our interviews negatively are not observed in our virtual fieldwork. When it comes to “age”, it is also well known that “younger people are more familiar with synchronous chat” (James and Busher 2016, p. 10). Considering gendered aspects of the online interviews, it is seen that numbers of females were more than males amongst the interviewees; the researchers being female themselves were aware of the gendered dimensions of spatial mobilities. There is always “a possibility that the gender difference between the researcher and the participant may have been the critical factor influencing rapport building” (Chiumento et al. 2018, p. 6). Under these conditions, we have had a self-reflexive and also a feminist approach (Yost and Chmielewski 2013; Grafanaki 1996) to our interviews whilst we attempted to create the conditions that are conducible to do the interviews in a quasi-real environment, enhancing trust and finding better listening techniques with each interview.

Online interviews require meticulousness as much as the face-to-face interviews. Deakin and Wakefield (2014, p. 604) suggest that the online interviews offer great flexibilities. On the other hand, t’Hart (2021) is of the idea that despite online interview is a great tool for research, “a layer of meaning” can be lost by the technological difficulties and which can affect the researchers’ capacity to be able to listen better. Since technology is involved, the interviews shall be in a place away from distractions (Deakin and Wakefield 2014, p. 609). Henceforth, before the interviews, the same rigorous process is visible. In sum, all this evidence suggests that the online interviews should not be considered as an easy option (James and Busher 2009, p. 9).

The blurring line between the researchers and the researched is used in grounded and constructionist methods in ethnography (Zavos and Biglia 2009; Cammarota and Fine 2008). Guided by these studies, rather than focussing on bias, the aim was to concentrate on the fact that there can be a way to diminish the hierarchies and power imbalances between the researcher and the researched. On the other hand, by not including the pilot interview results in our analysis we have avoided the bias in the data. However, the pilot interview was an interesting one for us to understand diverse aspects of online interviews, such as disruptive factors and factors that lead to a successful interview. In research, it can be noted that researchers themselves are instruments of research (Adu 2019, Kvale and Brinkmann 2015). Further, conducting pilot online interviews, it is very helpful “if the researchers can rehearse strategies to manage foreseeable differences in the experience of online interviewing” (Chiumento et al. 2018, p. 7). Therefore, to avoid the hierarchies and power imbalances and to rehearse different strategies, the online pilot interview was a sound beginning to the fieldwork.

## Setting the context for online interviews

The current literature mainly focuses on the advantages and disadvantages of the online interviews (Sedgwick and Spiers 2009; Salmons 2011; James and Busher 2012; Szolnoki and Hoffmann 2013; Johnson et al. 2019) but not sufficiently on preparing for online interviews. Moreover, most of the discussions about online interviews are written by departments such as nursing, psychology, psychiatry and medicine studies rather than sociology, political science, anthropology, ethnography and geography, for instance. Hence, it is observed that there is a gap in the literature that needs to be addressed by social scientists as well. Nevertheless, before addressing this gap, it is necessary to have a thorough overview of the literature, which postulates so many essential elements of online interviews.

“An interview is an information-gathering technique in which the defining feature is the presence of an interaction between the interviewer and the interviewee” (McCrady et al. 2010, p. 109). Interviews are valuable for the flexibility they provide to both sides, as well as high levels of information sharing despite their caveats (Atkinson and Silverman 1997). To assess the quality of the interviews, the researchers looked at three criteria: word count, interview duration and the presence of topic-related data (Johnson et al. 2019, p. 4). Before online interviews, telephone interviews and their differences from in-person interviews were discussed more often in the literature regarding qualitative research. Hence, the literature on phone interviews cannot be underestimated (Irvine 2010). For instance, in phone interviews, people use more verbal clues compared to in-person interviews (Johnson et al. 2019) and this can be an advantage for the researcher.

First of all, the literature makes clear that the preparation for online interviews should not be taken for granted. Salmons (2011, p. 11) states:

When operating online, greater clarity and precision is needed, since the potential for misunderstanding is arguably more significant. Researchers and research participants need to know what is expected of them, why, and when. Both need to be sure that when a consent agreement is signed, all parties are clear about the purpose of the study, the use of the data—and the parameters of the data collection.

If there is “greater clarity and precision” in the way the interview is realised, there are many advantages to interviews online. One of the benefits is related to how these interviews help the researchers reach out to those that they cannot access in extraordinary conditions such as Covid-19 pandemic. For sure, having a limited budget and not being able to conduct interviews in different countries, online interviews grant great opportunities to researchers (James and Busher 2012). There is an intensification of usage of this method due to the measures taken with the presence of Covid-19 that has changed the way people work and conduct research during smart-working.

The advantages and disadvantages to online interviews might not be evident at first sight, as it is also possible that depending on the interviewer or interviewee, the interview might produce the best or the worst results. What we see in Curasi’s (2001) work is that “the electronically conducted interviews contain the strongest and the weakest transcripts” (p. 4). Considering Curasi’s observations, we believe that a good interviewer will be able to generate information also on the online interview.

One of the essential advantages of online interviews is geographical and time-wise ease that it offers (Hamilton 2014; Shapka et al. 2016; Mirick and Wladkowski 2019). In line with the first advantage, the researcher can decide to conduct interviews online because participants are geographically dispersed and there is not a possibility to collect the information via telephone interviews (Sedgwick and Spiers 2009; Salmons 2011, p. 12). The financial benefits of online interviews are not negligible for both the interviewer and the interviewee regarding the geographical aspect: “travel, lodging and meal expenses” will not be a part of the interview process (Joshi et al. 2020, p. 2; Deakin and Wakefield 2014, p. 604; O’Connor et al. 2008). Besides, not travelling for every interview to a different place, environmental sustainability is another plus that cannot be disregarded (Joshi et al. 2020, p. 3).

The second advantage is that there is no pressure of presence, which means that the presence of the interviewer could have been more stressful (Weller 2017, p. 617). Research suggests that the online interview method can be less stressful compared to face-to-face interviews. Deakin and Wakefield (2014, p. 604) “assert that online interviews can produce data as reliable and in-depth as produced during face-to-face encounters”. In this sense, the efficiency of virtual fieldwork should not be underestimated.

One of the most important disadvantages is limited capacity to build a rapport in the online interviews. Since building rapport might be considered more complicated, the researchers need to be cautious about this aspect as well. The overall qualities for building rapport include being sensitive to certain factors such as “ethnicity/culture, age and stage of life, gender, socioeconomic status, religious preference, presenting problems, comorbidity, problem severity and your own biases” (p. 114). Assuming that these qualities are also of utmost importance in in-person interviews, we argue that the results of an online interview are not so different from the face-to-face interview if the interviewer does not recognise possible biases that s/he might have.

In line with the second disadvantage, it is no secret that trust and understanding are the keys to qualitative interviews. "For the qualitatively minded researcher, the open-ended interview offers the opportunity for an authentic gaze into the soul of another, or even for a politically correct dialogue where the researcher and researched offer mutual understanding and support” (Atkinson and Silverman 1997, pp. 304–305). The potential loss of intimacy between the interviewee and interviewer (Seitz 2016) might occur as a disadvantage. In some cases, people are reserved, and they do not perform well on online interviews. In other words, “…when the person is reserved or less responsive, the visual cues being absent, it might be harder to build rapport” (Deakin and Wakefield 2014, p. 610).

There are issues that we consider not as advantages nor disadvantages but complications. These difficulties can be related to not knowing how to use the technology, the necessary software and not having access to the internet. One drawback is that “some might not possess the right technology” to do online interviews (Joshi et al. 2020, p. 3). Some interviewers might want to use a particular programme for their interviews, and if the other person does not know how to use this programme, which can cause alienation on the side of the interviewee. It can also happen that internet connection does not allow the interviewee or the interviewer to conduct a decent conversation (if the video or the voice do not function well), the interview might need to be repeated (Deakin and Wakefield 2014, p. 611).

To sum up other complications of online interviews in comparison with face-to-face interviews, we developed this diagram emanating from the previous literature written on online interviews:

It must be noted that most of the studies finally underline that the best way of interviewing is face to face or a mixture of in-person and online interviews (Johnson et al. 2019). Although differences of opinion still exist, there appears to be some agreement that the online interviews are an excellent alternative to in-person interviews if they are conducted with utmost care. In line with these thoughts, we argue that the difficulties of online communication are balanced by the pros of these interviews.

As indicated in the literature review, not all the researchers find the remote interviews as the most efficient and the ideal ones, yet during the times of Covid-19, we all had to plan our interviews remotely. This meant that a considerable research process on preparation for online interviews had to be put in place. In this section, we have discussed the advantages, disadvantages and complications. Now we want to detail all aspects of the pilot interview, separating it into three categories: *before, during and after the interview*. Using this analytical categorisation, we will dissect the intricacies of preparation, practice and retrospection for online interviews and expect that our pilot interview experience will perpetuate further suggestions to the research community.

## A brief introduction to the primary research project

As indicated initially, we presume that the spatially mobile and migrant skilled youth have already access to online software for communication purposes since they transnationally contact with their families and networks on a regular basis. Therefore, knowing that there would not be any kind of issues regarding software literacy for communication, for our pilot interview, we could just focus on other elements of online communication. This analytical section focuses on three parts: “before the interviews” gives information on the preparation for the project interviews (not the pilot interview), “during the interview” detail is focussed on the pilot interview and finally, “after the interviews” part focussed on considerations of both project interviews (51 semi-structured online interviews) and the pilot interview (conducted by and between the researchers).

## Before, during and after online interviews: procedures and processes

### Initial stages of the research

In this section, we reveal some clues about our preparation process for the research project, rather than the pilot interview. There are many issues to consider before conducting online interviews. Interview questions, online tools, sampling, preliminary coding and pondering on being an outsider or an insider are of great importance in the pre-interviewing process.

The confirmation bias was eliminated due to the fact that when we did the pilot interview, we had been colleagues only for 2 weeks. So, before the pilot interview, we had only a few online meetings and we had never met face to face and our acquaintance was fresh. Therefore, we could not have had the bias of knowing the other one’s past experiences except having seen the CVs and scholarly articles. Hence, the information about each other was inadequate. The only bias could emanate from the fact that the interviewed was more hesitant to talk about bad experiences in the academic life as she was trying to impress her (new) colleague. Therefore, there was some bias involved but the interview was unstructured and it allowed the interviewed researcher to talk freely without time limitations. The questions were not the same questions asked in 51 interviews, only three or four of them were asked and the fact that the interview was unstructured, one topic paved the way for another theme so it was smoother, longer but less focussed. The only barrier was the language barrier as the interview was conducted in Italian and the interviewed did not speak Italian at an advanced level. This limitation hindered obtaining a major amount of information that the interviewee could have transmitted.

Interestingly, more than bias there was a gender sensitive approach as the interview was between two female researchers, both in their mid-careers, without any kind of gender hierarchy being present. Moreover, the interviewer had prior experience of interviewing highly skilled Italians abroad. The bias was much less as the nationality of the interviewed was not Italian but Turkish.

## In preparation of the interview questions

One of the most critical steps before conducting online interviews is the preparation of questions. If the practice fits an ideal criterion upon which the researchers can be sure about which software to use, which interview questions to ask, making decisions on preliminary coding, and scheduling interviews according to the time zones, the process will proceed smoothly. Moreover, at the backstage, where the definition of the research question, research methodology and sampling are decided, a significant preparation process is at stake.

Bryman (2016, p. 473) underlines the indispensable qualities of an interviewer as mentioned by Kvale (1996): “knowledgeable, structuring, clear, gentle, sensitive, open, steering, critical, remembering, interpreting” and he also adds “balanced and ethically sensitive” (p. 473). We believe that most of the definitions of these adjectives are quite clear. Despite the clarity, we deliver the meanings of these two adjectives to satisfy the reader’s curiosity: “structuring: gives purpose for the interview; rounds it off, asks whether the interviewee has questions” and “critical: is prepared to challenge what is said—for example, dealing with inconsistencies in interviewees' reply.” (p. 473). In preparation of the interview questions we had to consider diverse themes (such as work, translation, daily life, cognitive remittances, precarity, uncertainty, temporality) and the questionnaire was changed more than a few times.

Our theme for the research project being emigration of skilled youth, we divided the topics into three: “the background (past), work and daily life (present), and the future projections (return, stay or further mobility)”. Detailing the questions, we decided to prepare a matrix where there is the theme, code, the related dilemmas we need to ask about in the form of keywords. Corresponding literature was also placed in this matrix, although the related literature (presented in the form of a bibliography) could have been increased if the themes were kept too broad. To make sure that all questions of interest are asked, diverse ways of formulating these questions were added under the sub-questions’ category. Following the matrix, the interviews would cover all the research questions we need to answer.

## Online tools for the interviews

We also needed to discuss which tools to use online, and another question was: are we going to use the same tools for everyone or are we going to move according to the preferences of the interviewees? It was decided that we would use different tools, depending on the comfort of the other participant who is giving us their time and most detailed insights about their lives. Therefore, we would choose the programme they prefer so that they could also feel comfortable during the interview.

There are different types of online interviews and software. Joshi et al. (2020, p. 2) talk about benefits of interviewing in their article naming additional software: “Zoom (Zoom Video Communications, Inc., San Jose, CA), Skype (Microsoft Corp., Redmond, WA), Webex (Cisco WebEx LLC, Santa Clara, CA), GoToMeeting (LogMeIn, Inc., Boston, MA), or Google Hangouts Meet (Google LLC, Mountain View, CA) delivers several benefits over traditional on-site interviewing.”^2^ For our pilot interview, we used Microsoft Teams which allowed us to record the meeting. Apart from Microsoft teams, it is fascinating to think about the range of online tools of communication. Deakin and Wakefield (2014, p. 609) underline the advantages of the Skype interviews and how it is easy for the interviewee to give only her or his Skype name and no more information on privacy (except when the name corresponds to the Skype name). However, the anonymisation can also be evaluated immediately after the interview during primary coding.

Another critical point is to find a suitable place to interview for the interviewee: “a location free from controllable distractions” (Deakin and Wakefield 2014, p. 609) is needed for a better concentration during the interview. However, for spatially mobile skilled emigrants, which are the subjects of our research, this impediment does not pose a problem as they do smart-working during the Covid-19 process. We isolated ourselves as much as possible during the interview process.

In addition to these aspects, “data security” and consent online are of great importance. The ethics of the research department was contacted, the forms on ethics were filled, and privacy statements were prepared. The privacy statements are sent to each interviewee to read and sign before the interview. The ones who are willing to be interviewed generally send a digital signature signing that they understand and accept how the data is going to be used, how the privacy will be respected and if it is not respected they can contact the department of the university to signpost any misuse of their data or any other questions that comes to their minds during and after the interviews. These are already the minimum requirements that are agreed by the researchers and the interviewees. As Busher and James (2012) argue, any kind of threat to the confidentiality and anonymity of the participants posed by online interviews shall be measured by the researchers. Therefore, at any stage of interviews (preparation, interviewing and analysis) all due respect shall be shown on privacy and anonymisation.

## Sampling

Sampling is essential regarding representativeness as well as in defining the limits of the research question. Moreover, selection helps the researcher not to be dealing with an enormous amount of data whilst also affording the researcher the chance to identify the sample with specific characteristics (Robinson 2014; Robinson and Smith 2010). Discussing the time and geography wise limitations with Covid-19, we have decided that the sample size would be in total 60. However, it was discussed that being aware of the difficulties that might arise during the research process, the sample size might be altered in the future. Regarding the alteration of the sample during the research process, Robinson (2014, p. 31) suggests: “the other major practical reason for changing sample size is if the availability of the resources, funding, time or researcher manpower lessens or increases during a project.” It is also suggested that the sample size might be decided to be smaller if the theoretical saturation is reached (Strauss and Corbin 1998). “Saturation is defined by many as the point at which the data collection process no longer offers any new or relevant data.” (Dworkin 2012, p. 1319). Dworkin says that the saturation level depends on many factors, including if the researcher is experienced enough to understand that a sufficient number of interviews are reached (ibid). However, if there is a need to answer this question more concretely, it is possible to say that “a huge number of articles, book chapters, and books recommend guidance and suggest anywhere from 5 to 50 participants as adequate.” (Dworkin 2012, p. 1319).

Our sample was selected from the responses to the questions that we send via Google forms. Within these forms, we had a couple of preliminary questions (age, gender, which university they graduated from, where they lived and if they were willing to participate in our research and we were also asking them their email address and other contact details to arrange the interview). Within these forms, we also required their informed consent to participate in the research. Therefore, they declared that they had read the laws on privacy and how the data will be used by the researchers involved in the project.

## Being an insider to mobility patterns and being an outsider as a researcher

Salmons (2011, p. 17) introduces the long-lived debate between being an insider or an outsider during the research process. We could have found many of the interviewees via our networks due to our international links. However, we preferred, in the end, to send a standard text of invitation via Google forms, which would allow us to do random sampling. This kind of dissemination of invitation will enable us to reach those who would be willing to interview: we could then adopt the “outsider” approach, as our networks and surroundings could have been biased with further limitations, devoid of the diversity of a sample that is more representative in age, gender and background. Likewise, by doing our pilot (trial) interview online (conducted by the authors of this paper), we had developed a broader acumen to a common problem that has attracted our attention as researchers: “researchers may be inspired to study a topic because they understand the issue or need from a personal, as well as a scholarly perspective” (Salmons 2011, p. 17). In our cases, the issue was “personal” as well as “scholarly”. Therefore, our interview trial was an intersecting one between the etic and emic perspectives where we are both insiders and outsiders to the theme of “mobility”.

### Important issues to consider in conducting interviews

Hereby, we focus on the pilot interview that we conducted as both insiders and outsiders to spatial youth mobilities. Therefore, it is one of the most exciting parts concerning observations. Since our interview was unstructured, some comments and suggestions on choosing semi or unstructured interviews will be provided; later we will present our statements on “appearance and voice” during the online interviews and finally, we talk about our reflections on the sense of time during the online interviews.

## Unstructured online interviews: pilot interview as a case study

The methodology of the interview conducted between the researchers of this paper was more of an unstructured nature whilst the interviews with the highly skilled Italians living abroad were semi-structured. Salmons (2011, p. 20) attribute this definition to the unstructured interviews: “*Unstructured* interviews are used to collect data through what is essentially a conversation between the researcher and participant.” As Bryman (2016, pp. 466–467) suggests in the qualitative interviews “rambling or going off at the tangents are often encouraged—it gives insight into what the interviewee sees as relevant and important; in quantitative research, it is usually recorded as a nuisance and discouraged.” In our interview guideline, we asked the questions decided in the beginning but also let the rambling take its course during the interview.

Definition of the unstructured online interview is as such: “Unstructured interviews do not use any set questions; instead, the interviewer asks open-ended questions based on a specific research topic, and will try to let the interview flow like a natural conversation. The interviewer modifies his or her questions to suit the candidate’s specific experiences.”^3^ Nevertheless, some parts of the semi-structured interviews can remind the unstructured interviews. Previous research suggested that “unstructured interviews” might be preferable when the interviewer is “looking for a full description of a phenomenon”. In our case, to acquire more in-depth information, it has been necessary to hybridise semi-structured and unstructured.

*During the pilot interview*, the interviewee (one of the authors of this paper) felt that she was very comfortable and had ample space to talk about her mobility experiences (including the hindering and fostering factors to mobility). From time to time, however, she would stop and look at the interviewee to ask about what she thinks is more exciting or central regarding the interviews. Although both the interviewee and interviewer were in a casual attitude during the interview; the natural order of the conversational practices in face-to-face interviews was not present for the reason that in-person interaction and immediate follow-up questions were not always synchronous as in-person interviews. Furthermore, the interviewee could not observe some of the mimics and gestures when she was talking, as she was not looking at the camera and not directly to the interviewer.

Regarding in-person interviews, it is widespread to have two people looking at each other, interrupting, asking and conversing more spontaneously. Since the interviewer was an expert in online interviews, it was also possible for her to create spontaneity. It might be assumed that online interviews make space for much less distraction compared to in-person interviews. Still, during a face-to-face interview, the interviewed and interviewee might get distracted by the music, general noise in the café, wherever the interview is set up, and also by observing other people in a public space. As a result of this, in the pilot interview, the external interactions were reduced because “what could happen in a room?” Yet, the pilot interview was not devoid of distractions. For instance, at some point, the interviewee had to close the window because of the noise coming from outside, the interviewer had to change the place as “online interviews are not the most ergonomic ones” (Bertrand and Bourdeau 2010).

Additionally, the interviewee also left her chair to open the door to the cat that wanted to get out of the room. Therefore, even if there were only a few, there were still some distractions caused by external conditions. However, it was observed that generally, the pilot interview went very smooth.

The interview was more in the form of the informality of a conversation, where all the detailed data could have been extractable: macro-, meso- and microaspects for mobility (general, familial and individual reasons for mobility and migration, for instance). In addition to being biographically rich, it is full of explanations about the personal history of spatial mobility. Yet, there is much work for the researcher-interviewer to do, especially when there is a rich auto-biographical content, and when the biographies can be complicated and multi-layered. Moreover, they might also not be very much chronological depending on the teller. However, the interviewers are still ready to trace connections when the interviewee is talking about almost anything, in an unstructured interview. “The trace of a biographical interview is open, hidden and internalised”^4^ (Bichi, 2007, p. 75). Bichi (2007, pp. 75–76) says that it is open because it can take different directions, it is hidden cause it does not always function like an interrogation and the trace is internalised by the researcher, as s/he relies on her pre-memory somehow.

Last but not least, the outlines of the pilot interview were clear, whilst the details and the sub-questions were flexibly asked. One advantage of this interview was that it allowed plenty of space for biographically dense material to come to the fore. Despite this advantage, a disadvantage of the unstructured interviews might be the chaotic phase of the coding process. Yet again, if grounded theory is utilised, even one unstructured interview can be the beginning of an information-wise rich analysis (Charmaz 2007). After the unstructured pilot interview, we thought that it would be wise to conduct semi-structured interviews with the highly skilled Italians abroad.

## Voice and appearance

At the meta-level, the unstructured pilot interview provided fascinating and ample results. Despite this positive side, the interviews online have an artificial character in which the information collected would not benefit from all the senses of in-person interviews. Accordingly, both the interviewee and the interviewer noticed that online conversations produce some limitations regarding the voice and appearance as crucial elements.

First of all, it is observed that the sentiments are not easily noticed in the voice of the interviewee as the agent is often homogenised via the microphone. In an online interview, it is thought that the voice is more liberated than the in-person interview which is similar to the advantages of telephone interviews as indicated above and yet, it is not always the case that the programme registers the differences in the tones of the voices. In other words, the sounds are more direct and flatter. To catch the nuances in changing voice tones (according to the changing emotions) is difficult. Therefore, some of the relational aspects of the interviews and connections are slightly lost. Since qualitative researchers are mostly interested in why and how, and how the interviewee says certain things during the interview, the downside of this aspect is not negligible.

When it comes to appearance, it is not difficult to notice that there is a rectangular frame (like in a film shooting) that is available to the interviewee and interviewer within which the gestures can be seen partially. For instance, when someone wants to say something like “when I left my country, my child was this tall…” with a hand gesture; only a part of the hand can be seen, and not the height from the ground—on the contrary, doing in-person interviews, seeing all the gestures and mimics would have been much less of a problem, as the person is not in a “rectangle”.

## Time flies: the sense of time in an online pilot interview

One of the most insightful works is Burchi’s work (2008) on women who work from home and who establish all their work relations online whilst also practically working outside of the house. What happens when the house becomes the office? She rightfully says for the women working at home: “it is necessary to learn, as we see from the narratives, not to live ‘with the head inside the screen’, risking to lose the perception of one’s body at work without noticing the time flying.”^5^ In line with these words, during the days of smart-working and Covid-19 pandemic, we have apprehended that we will have to live our head inside the screen for a long whilst conducting online interviews. Yet, we also noticed that time flew in our pilot interview regardless of being inside or outside.

A very significant theme was the passing of the time. It was observed that the unstructured interview trial lasted 2 h and 10 min: The time perception, though, as indicated both by the interviewee and interviewer, was much shorter than the real time. This discernment, however can also emanate from the fact that the interviewer and interviewee had met before, online, conducted a couple of meetings as colleagues. A facilitating factor, in this sense, is that both researchers are used to being mobile and using online technologies to conduct interviews and keep connections with “home”. However, it should be noted that unstructured interviews and flying time can cause further discrepancies and difficulties during the analysis period. Therefore, some ideas to shift to semi-structured interviews have been discussed and confirmed in the aftermath of the pilot interview.

### Post-interview evaluation and observation

In this part, we focus on our post-pilot interview reflections. We observe the pilot interview experience and deliver some suggestions.

## Observing the registered interview

Observing this interview online, we have noted four most central points: (1) To keep track with time, information and reflections, (2) To be prepared with a questionnaire (semi-structured) and essential themes (if possible, codes as well) beforehand, (3) To take the time to think and formulate more ideas on the mobility experience, (4) To take measures not to lose synchronisation. These four points are essential to consider, and we will explain this one by one in this section.

As we have highlighted above, to keep track with time, the current information and a mix of reflections are difficult but necessary during the online interviews in general because the time flies literally. What’s more, the density of time might not be the same as it is for in-person interviews. This remark takes us to the second point: to have more efficiency within the time constraints where “all that is solid melts into air”; to keep the research question and the semi-structured questions in mind (and aside) is a must. When the interviewee is distracted, or when the rambling is less desired, to get the interviewee back into the track will be substantial within one-time online interviews.

Regarding the second point, if the interviewer is interested in finding what is “unknown” and what is “unasked”, “return to the matrix (with questions, codes and themes)” can be a desirable trait. As indicated before; to be prepared for the online interview with a matrix, questionnaire and some codes, gains even more importance.

The third element when we observe the interview is fascinating and indispensable: To take time to think and formulate more ideas on the mobility experience during the interview. However difficult it may seem, this is essential for a couple of reasons. Much of this requisite is caused by the fact that the interviewee can be carried away and the interviewer might not want to interrupt the interviewee. In such a dynamic, if the interviewee is not totally off the topic, most of the exciting ideas can also be born during the interviews. This would mean that if the time to reflect can be taken when listening to the interviewee, via active listening (taking notes, being attentive, asking follow-up questions); the mobility experience/story/narrative/biography might bring forward many other insights and theoretical connections.

We have also observed in this pilot interview that the synchronisation can be jeopardised from time to time during the pilot interview, where the interviewee and the interviewer can speak at the same time, they might not hear each other’s words, internet connection might fail, they might not both look at the camera, and yet the camera and the people’s faces are not at the same level and so on. Hence, many other elements contribute to non-synchronised behaviour, which means: when the whole interview quality is considered there are some discrepancies, and there is more monopolisation by the interviewee if the interview is not structured. Even if the interview aimed to be unstructured in the first place, some pre-interview notes handed to the interviewee before meeting would benefit both sides to gain direction and examine the themes profoundly.

Surprisingly, during the online interviews, having the camera across one’s face, one thinks that to talk continuously (till being interrupted) might be expected. Yet this element of non-interruption does not mean that the interview is going well. Consequently, there is a need to be “mindful” maybe even more during the online interview compared to an in-person interview. The time goes fast, and the interviewee might feel the need to fill the silences more. For this reason, maybe a short break of coffee, or a short break after 1 h (if not all the questions are answered) or just an informal stop to chat can help the interviewer to consolidate her notes, tick the items, see for herself/himself how the interview is proceeding. In the meanwhile, also the interviewee can gather her forces to reflect better and arrange her recollections in a more organised manner.

Our pilot interview trial was a relatively smooth one. The interview was recorded and was available to both. It was always possible to communicate via email to confirm non-confirmed issues, or misunderstandings or “fill in the blanks”. The same strategy would be followed for the online interviews in the context of the research project.

## 51 Interviews online

51 interviews with highly skilled Italians abroad were conducted online. We defined highly skilled as university graduates (specifically four universities situated in Tuscany). The interviews were realised via Zoom, Google Meets, and Microsoft teams and the main language was Italian. There were two researchers conducting the interviews, which are authors of this paper, one whose mother tongue is Italian and the other speaks upper intermediate level Italian. Whilst the non-Italian speaker asked the questions, the native Italian speaker took notes that would ease the transcription process afterwards.

In order to establish the ethical guidelines, the ethical committee of the University of Pisa confirmed the credentials at the onset of the research and we have also asked permission from the skilled Italians abroad for using a video recording or voice recording device. The ones on Microsoft teams were video recorded, whilst the ones on zoom and google meets were not but they were voice recorded.

The interviews were semi-structured as we wanted to allow some space to the skilled Italians to be able to express themselves freely and the topic not to be centralised so that interesting themes would come to the fore. The questions were related to why and how they went abroad, which was their first abroad experience, which job they were involved in, if they were socially and culturally integrated, if they felt that they would return to Italy or not, which are the advantages and disadvantages of having studied at an Italian (Tuscan in specific) university. Not all the questions were verbalised as some of the interviewees responded to multiple questions whilst answering to one question.

We have noticed that the online interviews can be as efficient as the face-to-face interviews. Sometimes the connection made it harder to understand what the other was saying but it was quite rare as we had a good internet connection and the interviewees also made sure that they had sufficient internet connections. The clarity of the screen was more of a problem than the clarity of the voice coming through the microphone. In some cases, the video was blurred and the appearance was not crystal clear.

The interviews took 1.5 h in average. The longest was 2 h 10 min and the shortest was 55 min. In general, the modality was as such that one asked the questions and listened, whilst the other took notes that were as accurate as possible. The one who made the questions also took notes, not to miss any information. The semi-structured nature of the interviews allowed the researchers to ask questions if there was a theme that was new and interesting, therefore, it was not a very rigid structure in which one only asked question and the other only took notes. After the finalisation of each interview, there have been a discussion of the results on what is new and what is interesting regarding mobility and migration of the highly skilled Italians.

These interviews were intriguing as they taught us many different ways of conducting interviews online. We have noticed that the pilot interview conducted between the two researchers was not biased but it had limitations, as the authors knew each other barely. Furthermore, in the first interview conducted amongst the 51 interviews, one of the researchers started the conversation without doing the small talk and introduction (Douglas 1985; Hammersley and Atkinson 2007) which did not help building rapport and generating responses from the side of the research participant. In the second interview, amongst 51 interviews, a good introduction was the main aim. As noted, the pilot interview would not let us observe this aspect because there was already familiarity to the theme by both researchers. It was well understood that, during online interviews, with people not connected before, it is very difficult to have a fluent conversation without a good introduction to the conversation (as good as the one that could be possible in real life face-to-face interviews).

## Concluding thoughts

In this paper, the pros and cons of online interviews together with possible complications and resolutions have been elaborated. Regarding our experience of online interviews based on our research background, the two scholars (referred as “we”) have conducted a pilot interview (an experiment of an online interview) to capture both the etic (as an outsider) and emic (as an insider) aspects of the research process. Before the interview, we elaborated on the research questions and interview questions for the main research project. During the pilot interview, we followed more of an unstructured interview method, not asking all the previously decided questions but leaving space for rambling. Lastly, after the interview, we examined all of these elements: organisation, time, the density of themes, mindfulness, and synchronisation. As a result of this reflection, and our interviews with 51 highly skilled Italians abroad, we also add sensitive issues that might emerge during the online interviews.

Our pilot interview experience replicates the findings indicating all the advantages and disadvantages of this method (Hamilton 2014; Shapka et al. 2016; Mirick and Wladkowski 2019; Sedgwick and Spiers 2009; Salmons 2011; Szolnoki and Hoffmann 2013; Johnson et al. 2019). Besides the advantages and disadvantages, this method turned out to be a very fruitful one during the Covid-19 pandemic. We contribute to the literature dissecting the way in which the diligence of doing these interviews is observed before, during and after interviews:

### Organisation

First of all, it is essential to organise for the online interview for both sides. To prepare mentally (with questions, matrix, themes, codes) in a full-fledged manner beforehand is compulsory, because of the perception of time that seems to be slower but it is in fact, much faster, during the online interview.

Online interviews offer great opportunities and although they might have some fatiguing moments (being exposed to a screen for a long time and keeping the attention on a camera frame), it is still an efficient method and much less costly. By fatiguing moments, we mean that preparing comfortable physical conditions before the interviews are crucial for both sides.

### Time

The time can be perceived as less than it is, and 2 h might run smoothly in an unstructured interview. Therefore, some structure is needed, and it is the interviewer’s talent to alternate between no structure and some structure that can be inserted spontaneously. This is one of the reasons why we decided to do semi-structured interviews online after the pilot interview experience.

### Density of themes

The researchers expect valid information and also generalisable findings from the interviews. However, they also hope to hear about singular, more biographically rich experiences and reflections. It is essential to use a language that limits the boundaries of the leading research questions whilst perpetuating possibilities to transcend the theme during the interview with flexibility and conversational attitude. Transcending the themes would be realised via active listening and taking notes, when necessary and when an interesting (never-heard-before) topic comes into the discussion. Last but not least, despite the difficulty to establish rapport, we have also found that there is a need to “be sensitive to body language” so that we can “establish rapport even when sharing the same physical space was not possible” (Sedgwick and Spiers 2009, p. 6).

### Mindfulness

We have seen that the camera adds great qualities to online interviews in comparison with telephone interviews. However, some significant elements that convey greater meaning are still lost as the camera has a specific spatial limitation. It is imperative, therefore, to find the balance between leaving space for freedom to the interviewee (as the online interviews have their parameters regarding voice/appearance and lack of intimacy because of lack of presence) since “the frame is the interviewee’s room” (Sedgwick and Spiers 2009). Yet, space is even more limited: what is seen and observed is only a part of the room. In this case, freedom with unstructuredness can be balanced by some follow-up questions to receive more profound answers. Additionally, it is also true that there are moments where one forgets that there is a camera which is one of the empowering sides of the video-interviews.

### Synchronisation

In the online interview, although there might be a perfect internet connection and efficient software, it might still be the case that synchronicity is damaged by a couple of factors: an interruption, a consumed battery, someone entering the room or a ringing phone. Hence, it is almost always desirable to remain in the context through which the interview is constructed. Yet, it is also a good idea to think that we are reliant on electricity and internet, the interviewer needs to consider the most accommodating circumstances. Synchronisation is about asking and answering questions catching the right moments; observing every mimic and gesture is highly recommended. In this case, recording the video meeting can be a perfect solution if the consent of the interviewee is validated.

### Sensitive issues

Additionally, we found that it is always safe not to open the boxes that cannot be closed with questions which are harder to answer. That’s to say, if there is a question that might lead to thought-provoking themes and more profound dilemmas, the interviewer has to decide (in advance) if s/he wanted to initiate the discussion or not. Cause once an issue is opened during the interview, the structure and freedom dichotomy can create tension. Opening a discourse not aimed before might lead to a private and emotional element that concerns the interviewee's inner life and this question/discussion/dilemma has to be evaluated carefully. In short, distance matters and if the interviewee feels that considering the distance, to open this theme can cause the interviewer to be a miner rather than a gardener or traveller (Salmons 2011), the interviewer might choose to avoid it.

Further agenda for research could be on these themes: how can we realise online interviews with those whose conditions are creating vulnerabilities? High-skilled migrants were the leading focus group of our paper, and we already mentioned that this group is privileged about online tools and comforts of being skilled in using technologies. What about those in detention centres, prisons, elderly care centres, migrants in refugee camps and migrants at the borders?

This paper aimed to shed light on the preparation for online interviews based on the observation of a pilot interview conducted by researchers amongst themselves. The fact that Covid-19 has dominated the way we do research, we have been thinking and elaborating on this theme for a while now. In sum, we suggest to the researchers to do the following as they prepare for online interviews: to design a matrix with some coding, to prepare preliminary semi-structured interview questions but still allow for improvisation-unstructuredness if needed; to keep track of time and be mindful to the themes; not to open emotional boxes that cannot be closed during the conversation, and to do a couple of pilot interviews to become aware of the features of this method. Every researcher has a diverse way of interviewing; to share the experiences is essential to ameliorate the conditions for conducting online interviews and retrieving data more respectably and reciprocally.

## Data Availability

In case needed, the data (online interview in video and sound) is available for this article.

## Footnotes

1 From this point on, in the paper, the “pilot interview” will be used in relation with the interview we conducted to try the system of online interviewing as an insider and outsider to the theme of youth mobilities in the context of skilled emigration. The “interviews” with skilled emigrants are related to our research project where all the interviews will be with the high-skilled emigrants abroad. Finally, when we talk about “online interviews” we want to underline that these are the interviews in a generalised context, synchronously conducted via software(s) by the research community.

2 Since their focus is job interviews online during the Covid-19, the article cannot be generalised to stretch into unstructured qualitative interviews as job interviews are quite structured most of the time if not semi-structured.

3 https://www.simplypsychology.org/interviews.html#unstructured accessed on 27th of July 2020.

4 “La traccia di un’intervista biografica è aperta, nascosta e interiorizzata.”.

5 “è necessario imparare, si Legge nei racconti, a non-Vivere “con la testa Dentro lo schermo”, rischiando di perdere la percezione del proprio corpo al Lavoro o Quella del tempo che passa, inavvertito.”
